# Defining the role of Lgr5^+^ stem cells in colorectal cancer: from basic research to clinical applications

**DOI:** 10.1186/s13073-017-0460-y

**Published:** 2017-07-18

**Authors:** Masayuki Fujii, Toshiro Sato

**Affiliations:** Department of Gastroenterology, Keio University School of Medicine, 35 Shinanomachi, Shinjuku-ku, Tokyo, 160-8582 Japan

## Abstract

Intestinal epithelium is structured by two distinct components: the villi and the crypts. The crypts harbor stem cells expressing Lgr5 and thus have been a representative model to study tissue stem cell functions. Recent advances in organoid technology and analytical modalities have enabled precise characterization of Lgr5^+^ intestinal stem cells, providing insights into their roles in homeostasis and cancer.

## Introduction

The intestinal epithelium is one of the most frequently renewing tissues in the human body, with a turnover period of 3–5 days in the small intestine. This rapid renewal of the intestinal epithelium is driven by the stem cells situated at the crypt bottom, providing an excellent model to study stem cell functions and dynamics. Since the identification of Lgr5 as a surface marker of intestinal stem cells (ISCs) [1], a growing body of research on the molecular and functional regulation of Lgr5^+^ ISCs has refined our understanding of their roles in homeostasis, as well as in tissue injury and tumor biology. Organoid technology has enabled the reconstruction and expansion of intestinal epithelial tissue and has become a powerful tool to investigate ISC functions in vitro and in vivo. For instance, human colorectal cancer (CRC)-derived organoids phenocopy histological and molecular traits of patient tumors under culture and upon xenografting. This property of patient-derived organoids as “living” proxies of individual patient tumors may accelerate personalization and optimization of chemotherapeutic strategies. Two independent groups recently developed metastasis-permissive mouse CRC models based on organoid technology, genome editing, and orthotopic allogeneic transplantation, which provide valuable systems allowing in situ replication of the entire CRC progression process from tumor initiation to metastasis [2, 3]. These recent technical advances have produced several novel insights into Lgr5^+^ ISCs, especially in their contribution to tumorigenesis.

## The roles of LGR5^+^ cells in colorectal cancer

Cancer stem cells (CSCs) are hypothesized to be a subpopulation of cancer cells with the unique ability to sustain tumor growth, and are believed to be responsible for tumor metastasis and relapse. Thus, identification, characterization, and targeting of CSCs have been investigated as a strategy for cancer therapy. Lineage-tracing experiments have shown that Lgr5^+^ cells act as stem cells in mouse adenomas; however, whether stem cell hierarchy is similarly active in intestinal cancers, especially in human CRCs, has been elusive. To date, the enrichment of such CSCs has been assessed by the limiting dilution reconstitution assay, where isolated tumor cells are sorted based on specific surface markers and evaluated for their tumorigenicity upon xenografting. This approach permits frequency estimation of the clonogenic cells within a tumor, but this requires disruption of the tumor architecture, which may unavoidably skew the fate of isolated cells.

**Fig. 1:**
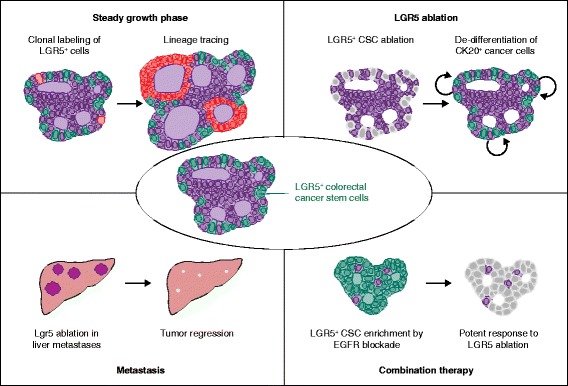
Context-dependent roles of LGR5^+^ colorectal cancer stem cells. The dynamics of LGR5^+^ colorectal cancer stem cells in different experimental settings. In intact tumors, LGR5^+^ cancer cells self-renew and their descendants generate differentiated cancer cells (*top left*), whereas upon LGR5 ablation, CK20^+^ differentiated cancer cells convert to LGR5^+^ cancer stem cells (*CSCs*; *top right*). In a mouse model of liver metastasis, Lgr5 ablation leads to complete elimination of metastatic tumors (*bottom left*). When co-treated with an EGFR antibody followed by LGR5 ablation, EGFR blockade induces enrichment of LGR5^+^ CSCs, thereby intensifying the effect of LGR5 ablation (*bottom right*)

To visualize directly the dynamics of LGR5^+^ cells in human CRCs, we recently devised an organoid-based method allowing genetic tracing of human CRC cells in xenografted patient-derived CRC organoids [4]. Lineage tracing of LGR5^+^ clones in tumor xenografts demonstrated that LGR5^+^ cancer cells have the ability to self-renew and generate differentiated progenies, thus functioning as CSCs in human CRC (Fig. 1, top left). From additional tracing experiments, we concluded that human CRC growth is mainly supported by LGR5^+^ CSCs but, upon their acute depletion, differentiated KRT20^+^ cancer cells dedifferentiate to stem cells to maintain the CSC pool (Fig. 1, top right). A similar dedifferentiation process can be observed in the non-tumor intestinal epithelium where, upon tissue injury such as radiation or colitis, early progenitor cells can revert to Lgr5^+^ ISCs to support tissue regeneration. CRC cells exhibited even more dramatic pliancy, as the fully differentiated cancer cells showed the potential to gain CSC capacity. Such functional flexibility of CRC cells contrasts with the rigid unidirectional hierarchy in healthy epithelium, which suggests CSC-targeting therapy alone may not be sufficient for the eradication of CRCs.

de Sousa e Melo et al. also investigated the roles of Lgr5^+^ cancer cells in mouse CRC models using organoids and genome-editing technology [5]. Consistent with our observations, ablation of Lgr5^+^ CSCs in orthotopically transplanted tumors suppressed tumor growth only during ablation. By contrast, Lgr5^+^ CSC ablation in liver metastasis models led to complete elimination of the metastatic foci (Fig. 1, bottom left). The specific mechanism behind the differing therapeutic responses of primary and metastatic tumors remains unknown; however, as the authors speculate, this may reflect varying induction of cancer cell plasticity between these tumor settings.

These works underline the potential efficacy of CSC targeting for CRC treatment but also illustrate the hurdle that cancer cell plasticity can pose in achieving satisfying therapeutic outcomes. Of note, we found that some CRCs undergo extensive volume regression when co-treated with an anti-EGFR antibody and LGR5 ablation. These tumors exhibited upregulation of LGR5 expression on anti-EGFR antibody treatment, implicating enrichment of LGR5^+^ CSCs by EGFR blockade (Fig. 1, bottom right). We surmise that such combination therapy may act synergistically by simultaneously targeting LGR5^+^ CSCs, non-CSCs, and possibly the dedifferentiation competency per se. Alternatively, targeting non-EGFR pathways may also modulate this process. Considering that the response to EGFR blockade is highly variable among CRCs, mainly depending on EGFR pathway mutations such as *KRAS*, *PIK3CA*, and *BRAF*, precise characterization of each tumor based on molecular signatures and phenotypes will be required for predicting response to the combination therapy. To this end, patient-derived organoids serve as a valuable resource allowing molecular profiling and functional validation of the potential targets or chemotherapeutics at a tumor level.

## Are Lgr5^+^ colorectal cancer cells heterotypic?

Cancers often exhibit substantial molecular diversity within individual tumors, and genetic dissection of this intratumoral heterogeneity has been intensively conducted through multiregional sequencing studies which provided novel insights into the trajectories of tumor evolution and the mechanisms of acquired resistance to molecular targeting therapies. Though it has been difficult to probe such molecular variation at a cellular level, recent advances in sequencing and microfluidic technologies have enabled high-throughput quantification of individual cell transcripts, providing single-cell-resolution snapshots of gene expression profiles from various tissues. Using single-cell mRNA sequencing, Barringa et al. recently identified Mex3a as a marker for a slowly dividing subpopulation of Lgr5^+^ ISCs that becomes highly proliferative upon tissue injury [6]. Notably, Mex3a^+^/Lgr5^+^ ISCs exhibited multipotency even in homeostasis, a distinct phenotype from another slow-dividing subpopulation of Lgr5^+^ cells that is committed to the secretory lineage [7], suggesting functional heterogeneity within the Lgr5^+^ compartment that can be revealed through transcriptomic profiling.

Given the heterogeneity found in homeostatic ISCs, parallel studies have investigated whether such heterogeneity exists in Lgr5^+^ cancer cells. Shiokawa et al. performed single-cell mRNA analyses on mouse CRC cells and non-tumor cells [8]. In their analyses, Lgr5^+^ intestinal cells clustered into four distinct populations. Interestingly, one of these subpopulations, which was characterized by downregulation of Ceacam1 and upregulation of Tcf1 long isoform, was uniquely observed in the tumor cells. These expression changes were functionally relevant in organoids, suggesting that the acquisition of these properties by Lgr5^+^ ISCs contributes to tumorigenic potential and that these molecules are potential therapeutic targets of CRCs.

In human colorectal tumors, recently Li et al. analyzed single-cell transcriptomes of multiple clinical CRC samples using a novel clustering method [9]. In this analysis, LGR5^+^ cancer cells converged into a unitary population; however, the transcriptional variability among LGR5^+^ cancer cells might have been diluted, as the principal aim of this study was to stratify promiscuous assembly of human CRC cells holistically. So far, functionally distinct subpopulations within human LGR5^+^ CRC cells remain unidentified, and enrichment and purification of LGR5^+^ cancer cells using LGR5 reporter organoids may facilitate discovery and functional assessment of potential human LGR5^+^ CSC subtypes. In light of substantial phenotypic heterogeneity among non-tumor Lgr5^+^ ISCs, it is tempting to anticipate the existence of a dormant LGR5^+^ CSC subgroup that can be mobilized upon therapeutic interventions such as radiation or chemotherapy, as demonstrated for Mex3a^+^/Lgr5^+^ ISCs [6]. These dormant LGR5^+^ CSCs may alternatively be resistant to such therapies and contribute to disease relapse or recurrence. Nonetheless, substantial intertumoral molecular diversity among human CRCs should also be considered, and analyzing tumors on an individual basis may allow a clearer delineation of the fractional transcriptomic heterogeneity among each subpopulation.

## An alternative metabolic niche modulates ISC identity

The fate determination process of tissue stem cells is strictly regulated by the surrounding niche environment. In the intestinal niche, Wnt, EGFR, Notch, and TGF-β/BMP are key pathways that regulate ISC homeostasis but are frequently hijacked in cancer by genetic mutations. As one of the niche components, terminally differentiated Paneth cells located adjacent to Lgr5^+^ ISCs support Lgr5^+^ ISCs by secreting niche ligands. In addition to this juxtacrine interaction, by using organoids and metabolomics Rodriguez-Colman et al. recently discovered that Paneth cells can also control ISC fate by dynamically supporting Lgr5^+^ ISC metabolism [10]. Specifically, they found that oxidative phosphorylation is distinctly active and essential in mature Lgr5^+^ ISCs and that glycolytic Paneth cells support oxidative Lgr5^+^ ISCs by supplying lactate. In view of this, the Lgr5^+^ ISC-specific metabolic profile may serve as an alternative hallmark of ISCs. Since Paneth cells are absent from colonic crypts, whether similar cell–cell metabolic communication exists in the colon or CRCs requires further investigation, but we believe that emergence of this new niche mediator will accelerate our understanding of Lgr5^+^ ISC regulation in these situations. As the authors document, anaerobic glycolysis, or the “Warburg effect”, is one of the hallmarks of cancer and may not only be an adaptive response to hypoxic cancer environments but contribute to maintenance of CSCs, as in the healthy epithelium. This speculation is in agreement with the relatively small proportion of clonogenic facultative LGR5^+^ CSCs in human CRCs [4], which suggests that the Warburg effect represents a majority of the differentiated CRC cells that potentially support LGR5^+^ CSCs. Thus, the hypoxic condition itself may be a supportive niche for the LGR5^+^ CSCs to thrive at hypovascular locations or metastatic sites.

## Closing remarks

Over the past decade, we have accumulated knowledge on how multifaceted signals from surrounding environments, as well as cell-intrinsic mechanisms, passively and actively coordinate the Lgr5^+^ ISC machinery. Nonetheless, further insights are of paramount importance, as the pathogenesis of numerous intestinal disorders, especially colorectal neoplasms, is attributed to disturbance of ISC function. The new technologies and discoveries documented above should facilitate our understanding of ISC regulation in healthy tissue as well as in diseases, potentially leading to establishment of therapeutic strategies directed toward the unique expression, signaling, or metabolic properties of these cells, as well as the identification of novel biomarkers.

## Abbreviations

CRC: Colorectal cancer
CSC: Cancer stem cell
ISC: Intestinal stem cell
